# Three-Dimensional Printing of a Spinal Interbody: Design Principles, Biomaterials, and Translational Considerations

**DOI:** 10.3390/jfb17030143

**Published:** 2026-03-12

**Authors:** Sahil Garg, Patrick Young, Christopher Franquemont, Rachel Conley, Sanjitpal Gill

**Affiliations:** 1 The Steadman Clinic, Vail, CO 81657, USA; 2 The Steadman Philippon Research Institute, Vail, CO 81657, USA; 3 School of Medicine, Loma Linda University, Loma Linda, CA 92350, USA

**Keywords:** 3D printing, interbody design, lumbar fusion

## Abstract

Background: Interbody spinal fusion is a common surgical treatment for degenerative, traumatic, and deformity-related spinal pathologies. Despite advances in cage geometry and fixation strategies that improve alignment and early stability, reliable fusion remains limited by the mechanical and biological constraints of conventional interbody implant materials. Traditional titanium and polymer-based cages often fail to optimally balance load sharing, osteointegration, and biological activity within the mechanically demanding interbody environment. This narrative review examines the development and translational potential of 3D-printed interbody fusion devices, with emphasis on how additive manufacturing enables the integration of mechanical performance with biologically active scaffold design. Methods: A thorough literature review was performed to evaluate the evolution, design principles, material properties, and translational outcomes of three-dimensional (3D)-printed interbody fusion devices. Results: Additive manufacturing enables precise control over implant architecture, allowing for the fabrication of porous, lattice-based cages with tunable stiffness, optimized load sharing, and enhanced bone–implant integration. Preclinical and early clinical studies suggest that 3D-printed porous titanium cages may reduce subsidence, promote osteointegration, and improve fusion-related outcomes compared with conventional designs. Emerging evidence indicates that scaffold porosity, surface microtopography, and bioactive coatings influence macrophage polarization, angiogenesis, and osteogenic signaling. Polymeric and composite constructs, particularly hybrid designs incorporating surface functionalization, represent promising adjuncts, though clinical evidence remains limited. Conclusions: Three-dimensional printing represents a paradigm shift in interbody fusion device design. Continued translational research and longer-term clinical follow-up are required to validate efficacy and guide widespread clinical adoption.

## 1. Introduction

Spinal fusion surgery involves joining two or more vertebrae to heal into a single osseous unit, with the goal of restoring stability, preserving neurological function, and sometimes alleviating pain caused by degenerative, traumatic, or deformity-related pathology [1,2]. Fusion has been demonstrated to improve outcomes in conditions such as spinal stenosis, degenerative and isthmic spondylolisthesis, a wide array of acute fracture patterns, and degenerative disk disease [3]. The fundamentals of treatment focus on the decompression of compromised neural elements and the restoration of stability between spinal segments. Each spinal segment consists of 2 adjacent vertebral bodies and their corresponding posterior elements. In many instances, to adequately decompress neural elements between segments, substantial bony resection may be required. In such instances which may compromise spinal stability, attempted fusion of the vertebral segments is required to restore biomechanical integrity. In this context, spinal fusion serves as a widely utilized tool to treat a variety of pathologies.

As part of the fusion process, an interbody device is commonly inserted between adjacent vertebral bodies at the level where the intervertebral disk is located (after prior removal). After placement, supplemental fixation using screw-based systems is utilized to further enhance stability of the spine during the initial processes of bone healing. These screw types may vary but grossly consist of either pedicle or lateral mass techniques based on the location of the spine (thoracolumbar and cervical, respectively).

The interbody cage itself plays a critical role in restoring disk height, maintaining segmental alignment, and facilitating biological fusion across the intervertebral space. In the short term, the device functions to distract the disk space and stabilize the spinal segment in a desired alignment (in combination with screw and rod fixation). Over time, its role transitions from supporting mechanical stability and segmental height to facilitating osteointegration and successful arthrodesis across the space [4]. Consequently, interbody cage design has become increasingly important as surgeons seek to optimize both alignment correction and fusion success.

From a biological standpoint, the interbody space represents a uniquely complex regenerative environment characterized by limited intrinsic vascularity, high mechanical demand, and close coupling between implant surface properties and host immune response. Unlike non-load-bearing bone regeneration sites, early cellular events within the interbody space are strongly influenced by local strain, implant stiffness, and surface microtopography, all of which modulate osteogenic differentiation and angiogenic signaling [5].

In recent years, interbody cage design has evolved to better address alignment and fusion goals. The evolution of cage design, materials, and placement technique has greatly advanced the efficacy of the lumbar interbody fusion procedure [3]. Novel geometries incorporating built-in curvature (lordotic cages) have improved sagittal alignment correction, while the incorporation of bone grafts and osteobiologics within and around cages has enhanced fusion potential [6]. Despite these advances, achieving reliable fusion remains challenging, and current interbody materials often fail to optimally balance mechanical stability with biological integration.

Emerging evidence suggests that fusion success is not dictated solely by bulk material selection, but rather by the interaction between implant architecture, surface characteristics, and the local biological milieu. Experimental work in interbody bone tissue engineering demonstrates that scaffold pore geometry and surface energy directly influence macrophage polarization, osteoblast recruitment, and vascular endothelial growth factor (VEGF) expression [5,7].

To achieve these objectives, surgeons may select from various interbody cage designs and materials [8,9,10]. Despite advances in implant technology, there remains an unmet need for interbody materials that provide mechanical stability while simultaneously promoting biological integration. Titanium and polyetheretherketone (PEEK) remain the most commonly used materials for spinal interbody implants [11]. Titanium exhibits a high elastic modulus relative to cortical bone, which may increase construct stiffness, reduce load sharing, and promote stress shielding, ultimately impairing long-term structural integrity [12]. In contrast, PEEK cages possess a lower elastic modulus but have bioinert surfaces that lack intrinsic osteointegrative properties [13]. This brief explanation highlights some of the challenges inherent in conventional interbody cage materials. These will be discussed at greater length subsequently.

Conventional fusion surgery is further limited by the inability of standard interbody implants to accommodate patient-specific anatomy and biomechanical demands. The transition from traditional implant design towards bioactive and patient-specific interbody constructs has demonstrated improvements in fusion and osteointegration rates [5]. Additive manufacturing, particularly 3D printing, enables the fabrication of interbody cages with controlled porosity, tailored mechanical properties, and complex geometries that more closely mimic native bone. Advancements in electrophoretic deposition (EPD) may be used in conjunction with 3D printing to coat 3D-printed interbody devices and augment desired physiological characteristics of biomaterials [14]. This review focuses on recent advances in 3D-printed interbody fusion devices, with particular attention to their material composition, structural design, and translational potential. While existing studies largely consist of preclinical models and early clinical data, emerging evidence suggests that 3D-printed interbody cages may achieve greater bone volume production, fusion rates, reduced segmental motion, and improved mechanical stability compared to conventional designs [15].

Additive manufacturing further enables deliberate control over scaffold architecture at the microscale, allowing for the modulation of mechanotransduction pathways and immune–osteogenic coupling within the interbody space. Unlike conventionally manufactured cages, 3D-printed interbody scaffolds can be designed to promote favorable macrophage phenotypes, enhance osteoblast activity, and support neovascularization through tailored pore size and interconnectivity, positioning additive manufacturing as a biologically active design strategy rather than a purely mechanical innovation [5].

## 2. Materials and Methods

A narrative literature review was conducted in accordance with the Scale for the Assessment of Narrative Review Articles (SANRA) guidelines [16]. A search of PubMed, Scopus, and Web of Science was performed using the following search strategy: (“Printing, Three-Dimensional” [MeSH] OR “3D printing”) AND (“Spinal Fusion” [MeSH] OR “interbody fusion”). The search was limited to English-language articles published through January 2026. Original research articles, systematic reviews, meta-analyses, and clinical studies were considered for inclusion. Studies discussing biomaterials (titanium, PEEK, polymers, composites, etc.), design parameters (porosity, lattice geometry), mechanical properties, osseointegration, or clinical outcomes of spinal interbody fusion devices were included. Case reports were excluded. Studies focused exclusively on non-spinal orthopedic applications of 3D printing were excluded. A supplementary snowball search strategy was employed, whereby reference lists of identified articles were reviewed to capture additional relevant studies not identified through the primary database search. Articles were selected based on their relevance to the design, biomaterials, manufacturing processes, and translational considerations of 3D-printed interbody fusion devices.

## 3. Results and Discussion

### 3.1. Historical Perspective: From Autograft Dominance to Synthetic and Composite Grafts

Historically, spinal fusion relied almost exclusively on autologous bone graft, which was long regarded as the gold standard due to its inherent osteogenic, osteoinductive, and osteoconductive properties. Early interbody fusion techniques utilized structural autograft blocks or femoral ring allografts placed within the disk space to restore height and promote arthrodesis [17,18]. While these approaches demonstrated acceptable fusion rates, they were limited by graft availability, donor-site morbidity, and variability in mechanical performance, particularly in load-bearing interbody applications [19]. These constraints became increasingly apparent as fusion techniques evolved toward multilevel constructs and revision procedures, where graft volume requirements exceeded what could be reliably harvested from the iliac crest [20].

In addition to donor-site morbidity, early structural grafts were limited by inconsistent load-sharing behavior and susceptibility to graft collapse or resorption under sustained axial loading, particularly in osteoporotic or multilevel fusion settings [5,21,22].

To address these limitations, the late twentieth and early twenty-first centuries saw a progressive transition toward synthetic and composite graft materials used within interbody fusion cages. This shift coincided with broader changes in spinal instrumentation, including the introduction of metallic interbody cages designed to provide immediate structural stability while serving as conduits for bone graft incorporation. Synthetic graft extenders and substitutes were developed to reduce dependence on autograft while maintaining an osteoconductive environment within the interbody space [21]. This transition reflected a growing recognition that successful interbody fusion required separation of mechanical and biological roles, with cages providing immediate structural stability and graft materials supporting subsequent bone regeneration [5].

Calcium phosphate-based materials, including β-tricalcium phosphate and hydroxyapatite, emerged as some of the earliest and most widely adopted synthetic graft options for interbody fusion [20]. These materials demonstrated favorable osteoconductive properties and were shown to support bone ingrowth when used within interbody constructs, often in combination with autograft or bone marrow aspirate [20]. However, their role remained primarily biological rather than structural, as these ceramics lacked the mechanical strength and fatigue resistance necessary to function as standalone interbody devices under physiologic spinal loading [23].

The limitations of both biologic grafts and early synthetic substitutes underscored a fundamental challenge in interbody fusion: the need for materials and constructs capable of simultaneously providing mechanical stability and a biologically optimal environment for fusion. This recognition catalyzed the development of composite interbody strategies, in which structural cages, initially machined metallic or polymer composite devices, were combined with osteoconductive or osteoinductive fillers. While these approaches improved fusion reliability, they also highlighted persistent mismatches between implant stiffness, load sharing, and host bone biology, setting the stage for the emergence of engineered, additively manufactured interbody devices designed to integrate mechanical and biological performance at the material level. Integration of scaffold architecture and surface bioactivity within a single construct appears therefore useful in an effort to overcome the historical trade-off between mechanical stability and biological integration [5].

### 3.2. Biological Requirements for a Successful Interbody Graft

Materials that constitute an effective interbody graft must satisfy three fundamental biological criteria: osteoconductivity, osteoinductivity, and osteogenicity. Osteoconductive materials provide a scaffold that supports cellular attachment, migration, and bone ingrowth but do not actively induce new bone formation. Osteoinductive materials stimulate the differentiation of progenitor cells into osteoblasts, thereby promoting de novo bone formation, while osteogenic materials contain viable cells capable of directly producing bone tissue. Together, these properties support the formation of a mechanically competent fusion mass capable of sustaining physiologic spinal loads [24,25].

Within the context of interbody fusion, these biological processes occur under continuous mechanical loading, which directly influences cellular differentiation pathways and extracellular matrix deposition. Bone tissue engineering studies specific to intervertebral environments demonstrate that mechanical cues synergize with biochemical signals to regulate osteogenic commitment and angiogenic activity, underscoring the need for graft materials that support both mechanotransduction and biological signaling [5,25,26].

In the interbody environment, these biological requirements must be satisfied within a constrained, load-bearing space where vascular access, mechanical stability, and implant–bone contact are tightly coupled [5,23]. As a result, the biological performance of an interbody device is influenced not only by graft composition but also by implant architecture, surface characteristics, and load-sharing behavior [3,5,27]. These considerations underscore the importance of interbody devices that integrate biological permissiveness with structural function, rather than relying solely on graft material packed within a mechanically inert cage. The ability of 3D printing to augment current design concepts to meet these biological needs will subsequently be discussed [3,5].

Importantly, these findings highlight that biological success in interbody fusion is not solely dependent on graft material selection, but on the coordinated interaction between scaffold architecture, mechanical environment, and host immune response, parameters that are uniquely addressable through additive manufacturing techniques [3,28].

### 3.3. Overview of Major Biomaterial Classes Used in Spine Fusion

One of the most challenging problems in the use of 3D printing in spine fusion is the proper identification of biomaterials that will not only be biocompatible and support osseous integration but also have a low likelihood of biological rejection while containing antiseptic properties so as not to cause infection [11,23,29,30]. Like their technological counterparts involved in regulating other organ systems, such as pacemakers or artificial teeth, spinal implants are also vulnerable to infection caused by various bacteria [31]. To prevent such complications, specific ceramics or polymers must be rigorously tested before they can be implemented in a spinal implant. Another set of properties that must be tested for biomaterials are their stress, strain, and deformation properties. The lifetime of a buried implant is evaluated by the unloading healing phase and the functional phase [32]. Failure in either of these tests would be catastrophic to the implant’s effectiveness and can become life-threatening to the patient.

Within the interbody environment, these requirements are magnified by sustained axial loading, limited vascular access, and intimate contact with vertebral endplates, where early immune responses to implanted materials strongly influence downstream osteogenic outcomes. Interbody bone tissue engineering studies demonstrate that implant surface chemistry and microarchitecture may modulate the inflammatory response that can either support or inhibit fusion biology [5].

In the context of 3D-printed spinal implants, the main material classes used revolve around metals and polymers which have been used to construct spinal implants that maintain strength and are able to withstand compressive forces without excessive stiffness [33,34]. Historically, other material classes, such as ceramics, have also been used for 3D-printed constructs but have fallen out of favor due to major obstacles in the literature involving low evidence showing a significant difference as well as author bias [34]. While ceramics continue to demonstrate favorable osteoconductive properties, their limited fatigue resistance and inability to accommodate physiologic spinal loading have largely confined their use to adjunctive or composite roles rather than standalone interbody devices [5,34,35].

Titanium is one of the most widely used metal materials for bodily implants and has significantly evolved over time due to its longstanding use. Specifically, titanium cages have progressed from solid metal blocks to porous structures with varying shapes and sizes that can closely mimic properties of bone while maintaining biomechanical stability [14]. While other metals such as stainless steel or cobalt–chromium can be used in spinal implants, they have been shown to have more issues involving strain and osteointegration than their mainstream counterpart. A high Young’s elastic modulus, poor corrosion resistance, and allergic inflammation disqualify stainless steel while cobalt–chromium can elicit an adverse immunologic reaction in addition to a high elastic modulus [34,36] (Table 1). Across material types, titanium has been often cited as the most favorable and studied within the scope of currently utilized 3D printed interbody materials [33].

The specific properties of titanium that have made it so successful in implants include its stress-shielding properties as well as its general ability to remain bioaccessible in the body [43]. Titanium has well-established osteogenic properties when enhanced by ceramic coatings and adverse immunologic reactions are infrequent compared to other alloys; therefore, titanium has been identified as one of the current safest and most effective biomaterials for implantation [39].

Polymers are another class of biomaterials that have been shown to have great potential in the realm of spinal implants due to their favorable bioproperties. Specifically, some polymers have bioresorbable properties that allow them to serve as temporary alternatives to permanent implants [43], while others such as PEEK offer long-term structural stability. Despite their widespread use, there are several challenges present in clinical practices, including long-term inflammation arising from inadequate biocompatibility, inconsistencies in biological function, late-stage failure from oxidative degradation, and difficulties in meeting personalized medicine requirements [44].

From a biological perspective, polymer-based interbody devices often exhibit limited intrinsic bioactivity, which may impair early cell adhesion and delay vascular infiltration unless augmented through surface modification or composite design strategies [5]. Although there are many downsides to the use of polymers in implantation, it must be noted that current research has shown promise for its future use. The development of a universally applicable surface functionalization method compatible with diverse polymer substrates should offer modular customizability along with cost efficiency that other biomaterials cannot yet offer [44].

### 3.4. Fundamentals of 3D Printing for Bone Tissue Engineering

#### Principles of Additive Manufacturing and Relevance to Interbody Fusion

Additive manufacturing, commonly referred to as 3D printing, enables the layer-by-layer fabrication of complex structures directly from computer-aided design (CAD) models [45]. Unlike subtractive manufacturing techniques, additive processes permit precise control over internal architecture, surface topology, and material distribution [27,46]. These capabilities are particularly relevant to spinal interbody fusion, where implant geometry, stiffness, and porosity must be carefully balanced to provide immediate mechanical stability while maximizing the propensity for biological fusion [27].

At the microscale, this control allows for deliberate modulation of mechanobiological cues that influence osteogenic differentiation, angiogenesis, and immune cell behavior within the interbody environment, factors that are increasingly recognized as critical determinants of fusion success [5,27].

In the interbody environment, additive manufacturing allows implants to function not merely as spacers, but as engineered scaffolds that integrate mechanical load sharing with osteointegration [12,27]. By enabling control over lattice architecture, pore interconnectivity, and material gradients, 3D printing offers solutions to longstanding limitations of conventionally manufactured interbody devices, including stress shielding, subsidence, and inconsistent fusion performance [46,47] (Table 2).

Bone tissue engineering investigations specific to interbody constructs demonstrate that scaffold anisotropy and graded porosity may spatially regulate strain distribution, which in turn modulates osteoblast activity and vascular ingrowth across the fusion interface [5,29,48].

### 3.5. Additive Manufacturing Technologies Relevant to Interbody Devices

#### 3.5.1. Selective Laser Melting (SLM/Laser Powder Bed Fusion)

Selective laser melting/Selective Laser Sintering (SLM), also known as laser powder bed fusion (LPBF), is a metal additive manufacturing process in which a high-energy laser fully melts successive layers of metal powder to create near-fully dense components directly from CAD models. Using titanium alloys such as Ti-6Al-4V, SLM enables the fabrication of interbody cages with complex lattice geometries, graded porosity, and patient-specific shapes that are not achievable through conventional machining techniques [46,49]. In a recently published systematic review which examined the clinical efficacy of 3D-printed titanium cages using SLM, 3 patients out of 78 were readmitted for reoperation in 2 years. Clinically significant quality of life improvements and VAS score improvements were observed [4].

Beyond structural advantages, SLM enables precise control over pore morphology and surface roughness, both of which have been shown to influence macrophage polarization and osteogenic signaling within interbody bone tissue engineering models [24,50]. In spinal fusion surgery, SLM has been widely adopted for the production of porous titanium interbody cages designed to reduce stress shielding and improve load sharing across vertebral endplates [46,51]. Preclinical and early clinical studies demonstrate that topology-optimized SLM-fabricated cages can achieve favorable mechanical behavior while supporting bone ingrowth and osseointegration within the intervertebral space [11,51]. Beyond spinal applications, SLM has been applied to the design and fabrication of patient-specific orthopedic implants with favorable pore distribution, inter-pore connectivity, and bearing capacity, further demonstrating the versatility of this platform for individualized load-bearing constructs [52]. Continued optimization of metal 3D printer hardware components, including powder-spreading mechanisms, may further improve print consistency and reproducibility for medical-grade implants [52,53,54].

#### 3.5.2. Electron Beam Melting (EBM)

Electron beam melting (EBM) is a powder-bed fusion technique that utilizes a focused electron beam to selectively melt metal powder layers within a high-vacuum environment. Elevated build temperatures and vacuum conditions reduce oxidation and residual stress, making EBM particularly well suited for producing porous titanium interbody implants with controlled microarchitecture [45,55].

Interbody bone tissue engineering studies suggest that the higher build temperatures associated with EBM may also influence surface oxide layer formation, which can alter early cell adhesion and osteogenic response [56,57]. EBM-fabricated porous titanium cages have demonstrated elastic moduli closer to that of native bone and improved osseointegration compared with conventional polymer-based cages in preclinical spinal fusion models. These findings support EBM as a viable platform for load-bearing interbody devices that prioritize both mechanical stability and biological integration [55,57].

#### 3.5.3. Fused Deposition Modeling (FDM)

Fused deposition modeling (FDM) is an extrusion-based additive manufacturing technique in which thermoplastic filaments are deposited layer by layer to create three-dimensional structures. While FDM offers low cost and broad accessibility, the mechanical strength of printed constructs is generally insufficient for permanent load-bearing interbody implantation [43,58]. Despite these limitations, FDM enables rapid prototyping of porous polymer scaffolds that serve as valuable platforms for evaluating osteogenic and angiogenic responses in interbody bone tissue engineering research [5,59]. In spine surgery, FDM is primarily utilized for patient-specific anatomical models used in preoperative planning, surgical education, and simulation. Prior studies have shown that FDM-printed lumbar spine models provide realistic training platforms for pedicle screw placement and procedural rehearsal, serving as accessible alternatives to cadaveric models [60,61]. There are some novel applications of FDM in interbody design which will be discussed in greater detail subsequently, relating to the evolution of PEEK cages.

#### 3.5.4. Stereolithography (SLA)

Stereolithography (SLA) is a vat photopolymerization technique in which ultraviolet light selectively cures liquid photopolymer resin to produce highly accurate, high-resolution structures. Although SLA materials lack the mechanical properties required for interbody implantation, the technology is widely used in spine surgery for anatomical modeling and fabrication of patient-specific drill guides due to its dimensional precision [62,63].

Cadaveric validation studies using SLA-generated cervical spine models and drill templates have demonstrated high pedicle screw placement accuracy, supporting the role of SLA in surgical planning and navigation rather than permanent implantation [64,65].

### 3.6. Scaffold Architecture and Porosity in 3D-Printed Interbody Devices

Porosity is a defining feature of 3D-printed interbody devices and plays a critical role in both biological fusion and mechanical performance. Interconnected pore networks facilitate bone ingrowth and vascular infiltration, creating a three-dimensional microenvironment that supports cellular migration, nutrient diffusion, and long-term osteointegration. In additively manufactured metallic lattice structures, this architecture promotes stable bone–implant interfaces essential for durable interbody fusion [12,48,66,67]. Further data demonstrates that pore sizes, within the range commonly achievable through additive manufacturing, optimizes osteoblast infiltration while supporting capillary formation, thereby accelerating transition from inflammatory to reparative phases of fusion [67].

Porosity also directly influences implant stiffness and load sharing. By adjusting pore size, geometry, and overall porosity, the apparent elastic modulus of materials such as titanium or polymer-based composites can be tuned to more closely approximate that of native bone. This reduces stress shielding and promotes physiologic strain transfer across the fusion segment, mitigating subsidence and improving construct stability [12,65,66,67,68].

Porous titanium lattices fabricated through SLM or EBM can be engineered to achieve elastic moduli closer to that of cancellous bone, reducing stress shielding while maintaining sufficient fatigue strength. Biomechanical studies demonstrate that porous titanium interbody cages exhibit improved subsidence resistance and load sharing compared with solid titanium or early-generation polymer cages [27,51,69]. Beyond mechanical benefits, porous titanium scaffolds have been shown to promote environments conducive to osteogenesis, reinforcing the role of porosity as a biologically active design parameter rather than a purely structural feature [5,70,71]. While in-vivo biomechanical testing studies are needed, preliminarily computational modeling supports a graded porosity configuration of design to best balance a modulation of material stiffness and osteogenesis [72,73].

#### Lattice Geometry and Architecture in 3D-Printed Interbody Devices

3D-printed porous titanium interbody cages have demonstrated favorable fusion outcomes in both preclinical and clinical settings. In an ovine interbody fusion model, Johnson et al. reported that additively manufactured porous titanium cages demonstrated positive fusion on biomechanical testing, microcomputed tomography, and histomorphometry, outperforming traditional PEEK cages in bony integration [74]. Clinically, Calek et al. reported a 97.1% fusion rate on computed tomography at one year in 100 patients (137 levels) undergoing ALIF and LLIF with 3D-printed titanium cages, with no revisions required for cage subsidence, migration, or pseudarthrosis [75]. Current commercially available 3D-printed interbody devices predominantly utilize proprietary lattice configurations. However, computational analyses have identified stress concentrations at strut–node junctions as a mechanical limitation of such designs, and the relatively lower surface to volume ratio of such architectures may provide less substrate for cellular attachment compared to alternative configurations [73,76]. Biomechanical testing involving repetitive load cycles are therefore advisable to monitor for fatigue failure.

Triply periodic minimal surface (TPMS) architectures have emerged as a promising alternative. TPMS structures are mathematically defined, continuously curved surfaces characterized by zero mean curvature at every point and the absence of surface self-intersection [27,73]. This eliminates discrete strut–node junctions and produces a more homogeneous stress distribution under physiologic loading [27,73]. Among TPMS configurations, the Gyroid and Schwarz Diamond architectures have received the most extensive investigation in bone tissue engineering [73,77].

TPMS-based scaffolds fabricated in Ti-6Al-4V have demonstrated mechanical properties within the range of native bone. Timercan et al. compared strut-based diamond and sheet-based gyroid lattices at porosities of 60–80% and reported stiffness values of 1.9–4.8 GPa with mechanical resistance of 52–160 MPa, exceeding that of vertebral bone [73]. Reshadinezhad et al. designed TPMS structures specifically for intervertebral lumbar cages and reported that gyroid and diamond configurations with 70–75% porosity achieved elastic moduli of approximately 9–16 GPa, approaching the elastic modulus of cortical bone (7–20 GPa), with biomechanically significant prevention of stress shielding based on the intrinsic ratio of the stress field to the yield strength of the constituent material [78]. These findings underscore that a fine balance between pore size and porosity must be considered when optimizing lattice structure, and alternating these variables alongside TPMS scaffold type may allow designers to optimize stiffness, strength, and torsional yield strength [78,79]. Beyond mechanical performance, TPMS architectures offer favorable fluid permeability for nutrient diffusion and vascular infiltration, with gyroid structures demonstrating up to ten-fold greater permeability than conventional grid-based scaffolds [73,76]. In vivo comparisons by Maevskaia et al. demonstrated that Diamond and Gyroid TPMS scaffolds exhibited significantly higher compression strength, bone ingrowth, and bone-to-implant contact compared with primitive and conventional lattice designs, supporting these two as the most promising TPMS architectures for future implants [77].

While preclinical data supporting TPMS architectures in bone regeneration are encouraging, direct clinical evidence evaluating TPMS-based interbody devices in spinal fusion remains limited. Current commercially available 3D-printed interbody cages predominantly utilize proprietary lattice configurations, and the translation of TPMS designs into clinical interbody applications represents an active area of investigation. Future studies comparing fusion performance, subsidence profiles, and long-term mechanical behavior of TPMS-based interbody cages against conventional designs in clinical cohorts will be essential to define their role in spinal fusion surgery.

### 3.7. 3D-Printed Biomaterials in Spinal Fusion

Titanium interbody devices represent the most clinically mature application of 3D printing in spinal fusion. Additively manufactured titanium cages combine high compressive strength with tunable porosity, allowing for the simultaneous optimization of load-bearing capacity and biological integration. Interbody bone tissue engineering studies indicate that porous titanium scaffolds enhance early bone–implant contact and angiogenic signaling compared with non-porous designs, contributing to improved fusion biology [5,67].

In the current landscape of implant design, PEEK has sometimes been clinically preferred to titanium due to its modulus of elasticity being more proximal to bone. The clinical significance of this has been extensively studied, as PEEK has repeatedly demonstrated lower subsidence rates when compared to non-3D-printed titanium interbody devices.

### 3.8. Clinical Applications and Early Outcomes

In 2023, a study by Alan et al. challenged this notion through an investigation of 3D-printed titanium cages with the introduction of greater porosity allowable due to the manufacturing capabilities afforded by 3D printing. In this study, after matching cohorts who underwent Lateral Lumbar Interbody Fusion (LLIF), they identified that 3D-printed porous titanium cages demonstrated a 19% lower subsidence rate when compared to PEEK cages across 97 vertebral levels [80]. Several others have demonstrated either reductions in subsidence or non-inferiority when comparing 3D-printed porous titanium cages to PEEK [42,47,81,82,83].

One group has gone so far as to design hybrid material PEEK and titanium 3D-printed cages which demonstrated lower subsidence rates compared to standalone porous 3D-printed titanium [83]. From a biological standpoint, increased porosity in these clinical constructs is hypothesized to facilitate earlier bone–implant contact and vascular infiltration, which may contribute to improved mechanical stability over time despite initial concerns regarding implant stiffness [5,66].

Early clinical studies evaluating 3D-printed titanium interbody cages in transforaminal lumbar interbody fusion (TLIF), posterior lumbar interbody fusion (PLIF), anterior lumbar interbody fusion (ALIF), and vertebral body replacement procedures report favorable fusion rates and acceptable subsidence profiles [84,85]. These findings are consistent with more updated reviews highlighting favorable outcomes across case series studies utilizing 3D-printed titanium cages in single and multi-level fusion procedures [33]. While long-term comparative data remain limited, early evidence supports the translational potential of porous titanium cages as structural fusion devices capable of enhancing osteointegration. Early improvements in fusion metrics may be driven by accelerated transition from inflammatory to reparative phases of healing within porous scaffolds, reinforcing the need for longer-term clinical follow-up to assess durability of these effects [5,28].

#### Imaging Considerations for 3D-Printed Interbody Devices

Postoperative imaging plays a critical role in evaluating interbody fusion, neural decompression, and hardware-related complications following spinal fusion surgery. Both computed tomography (CT) and magnetic resonance imaging (MRI) are routinely utilized in the postoperative setting; however, the diagnostic utility of each modality is directly influenced by the material composition and architecture of the implanted interbody device [86,87].

On CT, metallic implants produce artifacts primarily through beam hardening and photon starvation, which manifest as streak artifacts and signal voids that may obscure visualization of the spinal canal, adjacent soft tissues, and the cage–endplate interface [87,88]. Titanium-based interbody devices, while producing fewer artifacts than cobalt–chromium or stainless-steel constructs, still generate sufficient artifact to impair assessment of intra-cage bone formation and trabecular bridging [86,89]. In contrast, PEEK is radiolucent on both CT and plain radiographs, permitting superior visualization of intracage bone formation and the cage–endplate interface [86,89]. Due to this radiolucency, small metallic markers are typically embedded within PEEK cages to allow for the monitoring of implant position on conventional radiographs [86]. Iterative metal artifact reduction algorithms have demonstrated improvements in soft tissue visualization around spinal instrumentation on CT, though their capacity to enhance osseous structure assessment remains limited [88]. More recently, dual-energy CT and high kilovoltage protocols have been explored as additional strategies to mitigate beam-hardening artifacts associated with metallic implants [90].

On MRI, artifacts arise from magnetic susceptibility differences between metallic implants and surrounding tissue, resulting in signal loss, geometric distortion, and failure of fat suppression [87]. Titanium, while paramagnetic and generally considered MRI-safe, possesses sufficient magnetic susceptibility to produce clinically relevant artifacts that may impair visualization of adjacent neural structures and the bone–implant interface [91]. The severity of these susceptibility artifacts is directly related to implant mass and material density. Importantly, the porous architecture achievable through additive manufacturing may offer an additional pathway for artifact reduction. Carter et al. demonstrated that additively manufactured lattice structures in Ti-6Al-4V predictably reduce MRI artifact severity, with a direct correlation between artifact magnitude and relative material density. In their analysis, a porous lattice design applied to a representative implant geometry achieved approximately 10% artifact reduction compared to traditional solid material [91]. Building upon this principle, Seres et al. quantified the relationship between scaffold porosity and effective magnetic susceptibility, demonstrating a linear correlation across 3D-printed Ti-6Al-4V scaffolds with porosities ranging from 60% to 90%. Their findings indicate that highly porous implants possess sufficiently low effective susceptibility to be more amenable to routine MRI [92].

Clinical observations have supported these experimental findings. In a case involving revision surgery around a porous 3D-printed titanium cervical cage, van den Brink and Lamerigts reported that MRI afforded adequate visualization of soft tissue and neurological structures surrounding the implant, facilitating assessment of neural compression and surgical planning for the revision procedure. The authors noted that porous 3D-printed titanium minimized distortion on both MRI and CT compared to solid titanium constructs, enabling more detailed evaluation of both the fusion process and decompression of neural elements. Histological analysis of the retrieved cage further demonstrated complete osseointegration at two years, reinforcing the biological performance of porous titanium architecture [93].

Taken together, these findings suggest that the porous architecture inherent to 3D-printed titanium interbody devices may confer a dual advantage: promoting biological integration while simultaneously reducing imaging artifacts that have historically limited postoperative assessment of titanium-based constructs. While PEEK retains a distinct advantage in imaging clarity due to its radiolucent nature, the artifact reduction afforded by porous titanium narrows this gap and may represent a meaningful consideration in implant selection, particularly in clinical scenarios where postoperative MRI surveillance is anticipated. Further comparative studies evaluating imaging artifact profiles between 3D-printed porous titanium and PEEK interbody devices in clinical cohorts are warranted to better define the practical significance of these observations. Additionally, investigation into the effect of increasing porosity on balancing osteogenic and osteointegrative advantages with imaging-related optimization is needed, as the relationship between porosity levels that maximize biological performance and those that minimize artifact burden has not yet been clearly defined.

### 3.9. Role of Bioactive Coatings and Surface Modification

Although scaffold architecture and bulk material properties govern mechanical behavior, surface modification strategies play a critical role in modulating the biological response at the bone–implant interface. Bioactive coatings and surface treatments can transform otherwise bioinert substrates into osteoconductive or osteoinductive surfaces that promote early cellular attachment and long-term osseointegration [94,95]. Studies have quantified that surface roughness plays an integral role in the stimulation of osteoblastic activity [56,96,97].

Interbody bone tissue engineering research demonstrates that surface micro- and nano-topography can influence protein adsorption and immune cell adhesion, thereby shaping early inflammatory signaling cascades that precede osteogenesis [5,98,99]. In general, three types of surface treatments are used in spine interbody design: micro-arc oxidation/plasma electrolytic oxidation (MAO), laser surface texturing (LST), and chemical or electrochemical deposition. Plasma-based surface treatments, ceramic coatings, and micro-/nano-scale texturing techniques have been shown to enhance osteoblast adhesion while reducing bacterial colonization. Emerging strategies increasingly combine antimicrobial and osteogenic surface properties within a single implant, reflecting a shift toward multifunctional interbody devices that address both biological and mechanical failure modes [30,99,100]. Interbody bone tissue engineering studies further indicate that dual-function surfaces may reduce reliance on high-dose osteoinductive biologics by promoting endogenous osteogenic signaling through surface-mediated cell–material interactions [50,71,98,101].

Chemical deposition techniques have been utilized to remove unintended consequences of SLM. SLM may sometimes produce undesired residual unbonded powder during manufacturing, which may compromise biological performance of the implant. This process-purity consideration is amplified in additively manufactured porous and lattice-based interbody cages, where complex internal architectures and microporous surfaces can harbor residual, partially fused or unmelted powder within porous networks that may be difficult to fully remove and verify using standard post-processing and inspection workflows [54]. Residual powder retention raises process purity concerns beyond surface quality alone, because retained powder and particulate at the bone–implant interface can adversely influence local immune responses, thereby potentially impairing osseointegration [54]. Such particulate debris is clinically relevant because metal-derived debris can promote macrophage-mediated inflammatory signaling and has been associated with adverse local tissue reactions, including metal–debris reactions and metallosis-type changes in orthopedic implant settings, potentially compromising the local biological environment required for stable osteointegration and fusion [102,103]. Accordingly, mitigation strategies typically emphasize post-processing and cleaning workflows intended to minimize retained powder burden in porous constructs (e.g., depowdering and surface finishing) and verification practices to assess porous regions for residual particulate and surface condition, particularly in devices with high porosity or graded lattice architectures [54]. The clinical significance of this is still poorly understood, the authors found no significant body of published work that discussed the processing purity of various on-market devices.

In efforts to utilize coating technology, some groups have attempted to coat PEEK with titanium. In vivo studies suggested significant enhancement in biocompatibility and bone conductivity. In a systematic review of clinical studies by Li et al., this coating was demonstrated to significantly improve fusion rates in the lumbar spine at 6 months. There did not appear to be a significant difference in subsidence rates, suggesting that titanium coating did not grossly alter the modulus of PEEK [44,104].

## 4. Polymeric and Composite 3D-Printed Interbody Devices

In recent years, attempts at introducing porosity and surface coatings to PEEK have been made in efforts to improve osseous integration [105]. This biomaterial-related concern in the context of fusion has been described to be a function of its hydrophobic nature [106]. FDM has recently allowed for the fabrication of fully interconnected porous PEEK scaffolds with pore sizes (≈100–600 μm), porosity (~60–70%), and lattice geometry closely resembling cancellous bone.

Through the use of FDM, porous PEEK implants both with and without HA coatings have been tested in preclinical trials, suggesting that increased porosity aids in stimulating greater osteoblast differentiation, osteogenesis, and expression of angiogenic factors such as vascular endothelial growth factor (VEGF) when compared to non-porous devices. While the compatibility of polymeric materials to stimulate a more favorable biological environment through 3D printing appears feasible, further research is required to develop products as efficacious as titanium across these dimensions. Nevertheless, in a preliminary study of 13 cervical spine levels, significant fusion capacity was described using CT imaging between 6 and 9 months postoperatively with no signs of subsidence. Bone tissue engineering data suggest that polymer-based constructs may benefit most when incorporated into hybrid designs that combine favorable mechanical properties with biologically active surfaces or fillers [11,107,108].

While promising, further study directly comparing the quality and speed of fusion between 3D-printed polymeric and titanium implants is advised to better define the role of additive manufacturing in augmenting the clinical efficacy of these biomaterials. Table 3 includes the key clinical trials which relate to 3D-printed interbodies. (Table 3).

### 4.1. Emerging Frontiers: Biofunctional and Bioprinted Interbody Constructs

Emerging strategies aim to integrate biologically active components directly into interbody devices. These include biofunctional scaffolds incorporating stem cells, growth factors such as BMP-2 or VEGF, extracellular vesicles (exosomes), or gene-activated matrices [110,111,112]. Interbody bone tissue engineering may allow for controlled spatial and temporal presentation of bioactive agents within printed scaffolds to enhance osteogenesis while mitigating the risks associated with supraphysiologic dosing.

Augmentation of devices through 3D printing potentially affords novel applications for localized drug delivery, particularly in the setting of infection or when sustained release of therapeutic agents is required.

### 4.2. Concerns

While preclinical studies demonstrate promising osteogenic and angiogenic effects, translation to clinical interbody fusion remains limited by regulatory, manufacturing, and safety considerations.

Mechanical evaluation of intervertebral body fusion devices is often benchmarked against consensus standards, including ASTM F2077 for static and fatigue testing (e.g., axial compression, compression–shear, and torsion under cyclic loading) and ASTM F2267 for subsidence assessment using vertebral body/endplate analogs under static axial compression [113,114]. Current biomechanical testing standards may inadequately replicate in vivo loading conditions, underscoring the need for more physiologically relevant validation models [114,115]. Most clinical studies examining differences in 3D printing and coating-based augmentations of interbody design are limited by 1-year follow-up. Interbody bone tissue engineering research further notes that short-term fusion metrics may not fully capture long-term scaffold remodeling, fatigue behavior or immune-mediated responses [116]. Complex porous architectures present unique sterilization challenges that may alter mechanical or surface properties [117]. Regulatory classification of patient-specific 3D-printed implants varies by jurisdiction, with evolving FDA and CE guidance impacting clinical adoption [117,118,119]. In the United States, most 3D-printed interbody fusion devices have been cleared through the FDA’s 510(k) pathway, which requires demonstration of substantial equivalence to a legally marketed predicate device rather than independent evidence of safety and efficacy. While this pathway has facilitated relatively rapid market entry for devices from multiple manufacturers, it does not mandate prospective clinical trials, raising questions about the adequacy of preclinical bench testing alone for novel porous architectures with no direct predicate. Post-market surveillance through the FDA’s Manufacturer and User Facility Device Experience (MAUDE) database and mandatory Medical Device Reporting (MDR) remains essential for capturing adverse events, including subsidence, migration, and implant failure, that may not manifest within the timeframe of premarket evaluation. Current 510(k) clearance numbers and MDR reports are publicly accessible through the FDA’s online databases and represent a valuable resource for clinicians evaluating specific commercially available devices.

### 4.3. Future Directions

Future interbody fusion technologies are likely to incorporate imaging-based patient-specific design, computational modeling, and AI-assisted prediction of fusion success. Integration with navigation and robotic systems may further enhance precision placement and outcomes.

Toop et al. demonstrated significantly lower subsidence rates with 3D-printed porous titanium compared to solid titanium cages in TLIF (5.5% vs. 24.4%, *p* = 0.001) [112]. Preclinically, the absence of autologous bone graft markedly reduced fusion rates in 3D-printed titanium cages (33% vs. 80–100%, *p* < 0.05), underscoring that scaffold architecture alone cannot substitute for biological grafting [120]. Importantly, no clinical study has systematically compared fusion rates across different 3D-printed lattice geometries—including variations in surface modification, coating, porosity, and lattice structure—relative to solid titanium cages. Such comparative clinical studies are advisable to determine which specific design parameters drive clinically meaningful differences in fusion and subsidence outcomes. Prospective, multicenter trials with minimum 2-year follow-up comparing 3D-printed titanium, coated PEEK, and composite interbody devices across standardized fusion assessment protocols represent a critical next step. Recent biomechanical evidence suggests that cage surface area and endplate topography, rather than porosity or bulk stiffness alone, may be the dominant implant-related determinants of subsidence, highlighting the need for design optimization studies that systematically isolate individual structural parameters [121].

Interbody bone tissue engineering paradigms increasingly emphasize patient-specific mechanobiological optimization, suggesting that future implants may be designed not only for anatomy but also for individualized healing capacity [5,122]. Maximization of the properties of biomaterials in combination with deformity-related fixation in design through the introduction of lordotic and patient-sized implants may confer significant clinical advantage.

## 5. Conclusions

3D printing has transformed interbody fusion by enabling implants that integrate mechanical stability with biological function. While gaps remain between preclinical innovation and widespread clinical adoption, continued advances in material science, manufacturing, and translational validation position 3D-printed interbody devices as able to redefine paradigms in spinal fusion surgery.

Collectively, insights from interbody bone tissue engineering reinforce that the success of future interbody devices will depend on harmonizing scaffold architecture, surface bioactivity, and immune modulation. Further study involving multiyear follow-up studies evaluating the clinical efficacy of these devices is advisable to establish the durability of current translational perspectives.

## Figures and Tables

**Table 1:** Elastic modulus of spinal interbody implant materials and native bone tissue.

Material	Material Class	Elastic Modulus (GPa)	Notes
Vertebral cancellous bone Cortical bone	Native tissue Native tissue	~0.1–0.5 [37] ~18 [38]	Mechanical reference for implant design in lumbar spine surgery Stiffness target for modulus-matching implant design
Solid titanium (Ti-6Al-4V)	Metal	~110 [38,39]	High modulus mismatch; associated with stress shielding and bone resorption
Porous titanium (3D-printed, SLM)	Additive metal	~3 [40]	Tunable modulus via lattice design; supports osseointegration; modulus varies by architecture
Stainless steel (316L)	Metal	~200 [39]	Highest modulus mismatch; largely obsolete in spinal interbody applications
Cobalt–chromium alloy (CoCr)	Metal	~210 [39]	Very high modulus; adverse immune responses reported
PEEK (solid)	Polymer	~3–4 [38]	Nearest bulk polymer modulus to cortical bone; bioinert surface limits osseointegration
Porous PEEK/HA composite (3D-printed)	Additive polymer composite	~0.05–0.6 [41]	Highly compliant; enhanced bioactivity from HA; modulus approaches cancellous bone
PEEK-Ti hybrid (3D-printed)	Additive polymer-metal	~3–4 [38,42]	Lower subsidence than standalone 3D-Ti cages; combines PEEK modulus with Ti surface osseointegration

Abbreviations: PEEK, polyetheretherketone; HA, hydroxyapatite; Ti, titanium; CoCr, cobalt–chromium; SLM, selective laser melting; Al, aluminum; V, vanadium.

**Table 2:** Comparative overview of elastic modulus, porosity, and stress shielding risk by implant material class.

Material	Elastic Modulus (GPa)	Porosity	Pore Size	Stress Shielding Risk
Solid titanium	~110 [38,39]	0%	N/A	High
Stainless steel	~200 [39]	0%	N/A	Very high
CoCr alloy	~210 [39]	0%	N/A	Very high
PEEK (solid)	~3–4 [38]	0%	N/A	Low
Porous Ti (3D-printed)	~3 [40]	Variable	Variable	Low to moderate
Porous PEEK/HA (3D-printed)	~0.05–0.6 [41]	60–70% [40]	100–600 µm [40]	Low
PEEK-Ti hybrid	~3–4 [38,42]	Variable	Variable	Low

Abbreviations: PEEK, polyetheretherketone; HA, hydroxyapatite; Ti, titanium; CoCr, cobalt–chromium; SLM, selective laser melting. N/A; Not applicable as material is solid.

**Table 3:** Summary of Registered Clinical Trials (ClinicalTrials.gov) Evaluating 3D-Printed Interbody Fusion Devices in Spinal Surgery.

#	NCT Number	Study Title	Material	Procedure	Comparator	N	Status	Outcomes
1	NCT05182489	Adaptix™ vs. PEEK Cages	Medtronic Adaptix™ 3D-printed porous Ti (Ti-6Al-4V)	1–2 level TLIF	Medtronic CAPSTONE^®^ PEEK	Up to 100 ^a^	Completed/Published [109]	100% fusion (BSF-3 ^b^) at 6 mo (3DPPT) vs. 0% (PEEK), *p* < 0.001. No significant difference in back pain, leg pain, or pain interference scores between groups [109].
2	NCT03647501	Nexxt Matrixx™ vs. PEEK Cages	Nexxt Matrixx™ 3D-printed porous Ti	1–2 level lumbar fusion	Honour™ PEEK	53 (25 Ti, 28 PEEK)	Completed/Published [2]	At 6 mo, BSF-3 ^b^ fusion in 27/34 levels (79.4%) Ti vs. 10/40 levels (25.0%) PEEK [2]
3	NCT04086784	3D-Printed Ti vs. PEEK in Osteoporotic PLIF	3D-printed porous Ti alloy	PLIF	Conventional PEEK	NR	Recruiting	Results pending
4	NCT05696470	DePuy Conduit™ in 3–4 Level ACDF	DePuy Conduit™ 3D-printed Ti	3–4 level ACDF (C2–T1)	Retrospective milled allograft	Up to 58	Active	Results pending
5	NCT05114356	TIDAL Cervical Device Registry	TIDAL 3D-printed Ti cervical interbody	ACDF	None (single-arm)	NR	Active	Results pending

Abbreviations: Ti, titanium; PEEK, polyetheretherketone; RCT, randomized controlled trial; TLIF, transforaminal lumbar interbody fusion; PLIF, posterior lumbar interbody fusion; ACDF, anterior cervical discectomy and fusion; BSF, Brantigan–Steffee–Fraser; 3DPPT, 3D-printed porous titanium; NR, not reported. **Footnotes:** ^a^ Study terminated early at interim analysis due to statistically significant results. ^b^ BSF-3: Brantigan–Steffee–Fraser Grade 3, defined as radiographic fusion with solid bone bridges within at least half of the fusion area.

## Data Availability

No new data were created or analyzed in this study. Data sharing is not applicable to this article.
